# Transesophageal echocardiography uncovers iatrogenic liver injury during minimally invasive mitral valve surgery: case report

**DOI:** 10.1186/s12871-025-03591-0

**Published:** 2025-12-30

**Authors:** Toshifumi Yanagi, Patrick Hussey, Kenichi Ueda

**Affiliations:** 1 Department of Anesthesiology, Kameda Medical Center, Kamogawa, 296-8602 Japan; 2 Department of Anesthesiology and Perioperative Medicine, The University of Alabama at Birmingham, Birmingham, AL United States

**Keywords:** Liver injuries, Minimally invasive surgical procedures, Cardiac surgical procedures, Echocardiography, Transesophageal, Cardiopulmonary bypass, Laparoscopy, Case reports

## Abstract

**Background:**

Minimally invasive cardiac surgery (MICS) reduces surgical trauma but limits direct visualization of surrounding structures, posing a risk of unrecognized injury.

**Case presentation:**

A 65-year-old man with alcoholic cirrhosis underwent mitral valvuloplasty, tricuspid annuloplasty, and Maze procedure via right mini-thoracotomy. During cardiopulmonary bypass (CPB), progressive reservoir volume loss was noted by a perfusionist. However, no collection of blood was evident in the surgical field, and thus, reservoir volume was maintained with volume transfusion. After weaning from CPB, transesophageal echocardiography (TEE) revealed free fluid around the liver. Exploratory laparoscopy confirmed a 1-cm liver laceration likely caused during a trocar insertion. Hemostasis was achieved surgically, and the patient recovered without complications.

**Conclusions:**

Iatrogenic liver injury during MICS is rare and may lack intraoperative hemodynamic signs. In this case, timely intraoperative detection of abdominal bleeding using TEE enabled immediate intervention. Routine TEE assessment of intra-abdominal free fluid may help identify similar injuries early and improve patient outcomes.

## Background

Minimally invasive cardiac surgery (MICS) is increasingly performed, particularly for mitral valve repair or replacement. It offers advantages such as reduced bleeding and shorter intensive care unit (ICU) and hospital stays compared with sternotomy [1]. However, due to limited visualization and restricted access to the surgical field, MICS carries its own risk of complications [2, 3]. Trocar placement carries inherent risks, including diaphragmatic injury, hepatic laceration, or injury to adjacent abdominal structures. We present a case of liver injury caused by the insertion of a trocar for an endoscopic camera during MICS, which was promptly identified using transesophageal echocardiography (TEE). 

## Case presentation

Written informed consent for publication of this case report was obtained from the patient, in accordance with institutional policies and ethical guidelines. This case report adheres to the CARE guidelines.

A 65-year-old man with a history of hypertension and alcoholic liver cirrhosis (Child–Pugh class A) was admitted to hospital. On presentation, he reported shortness of breath and palpitations, and was found to have paroxysmal atrial flutter without hemodynamic instability. Physical examination revealed a grade 3/6 systolic murmur at the apex. TEE demonstrated severe mitral regurgitation (MR) with an extensive prolapse of both the anterior and posterior leaflets. TEE also showed moderate tricuspid regurgitation (TR) with a dilated tricuspid annulus. Wall motion of the left ventricle was normal with an ejection fraction of 71%. Right heart catheterization revealed a mean pulmonary arterial pressure of 42 mmHg and a pulmonary capillary wedge pressure of 30 mmHg.

**Fig. 1:**
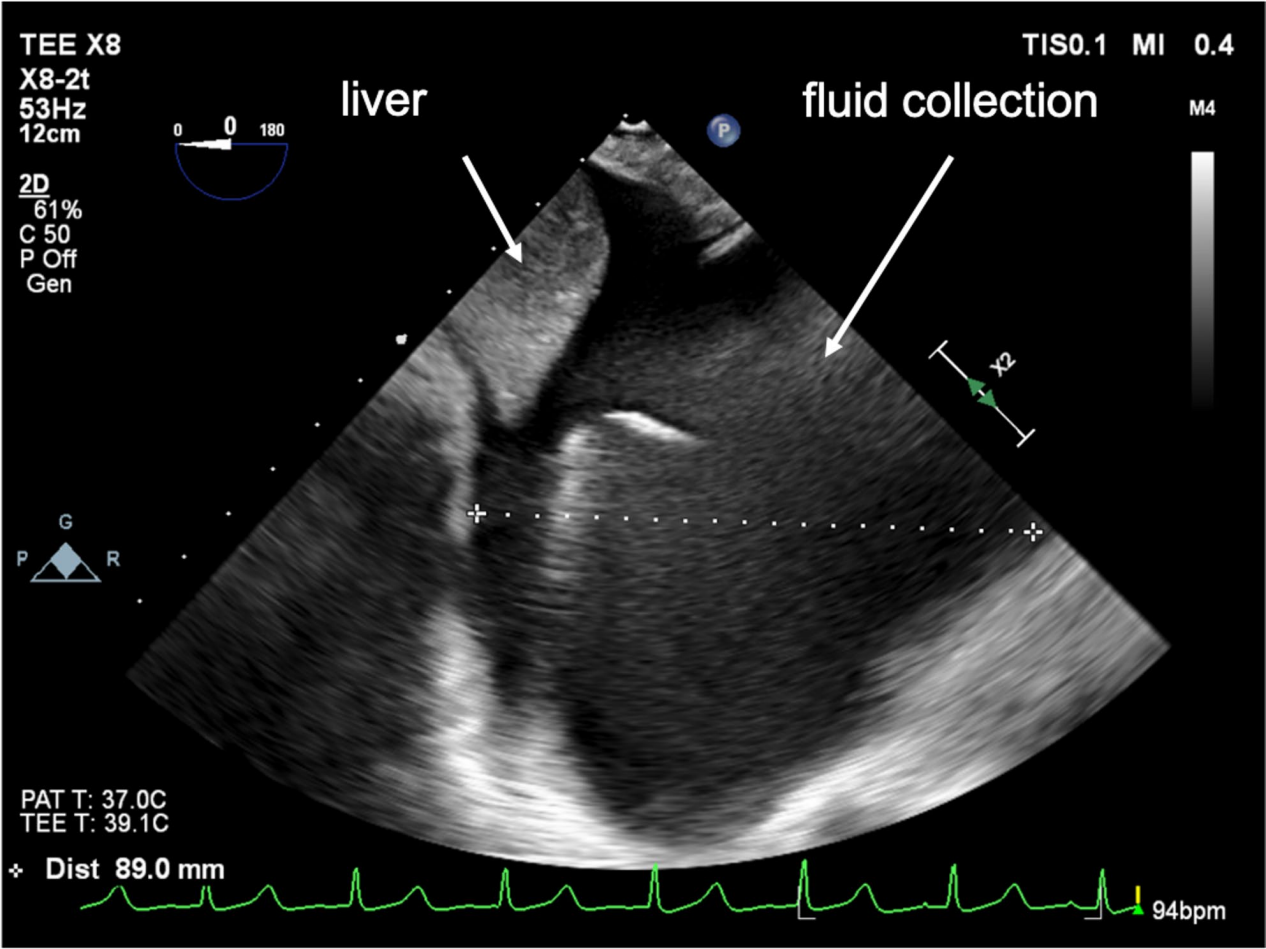
Deep transgastric TEE view demonstrating a hypoechoic space around the liver, consistent with intra-abdominal free fluid

**Fig. 2:**
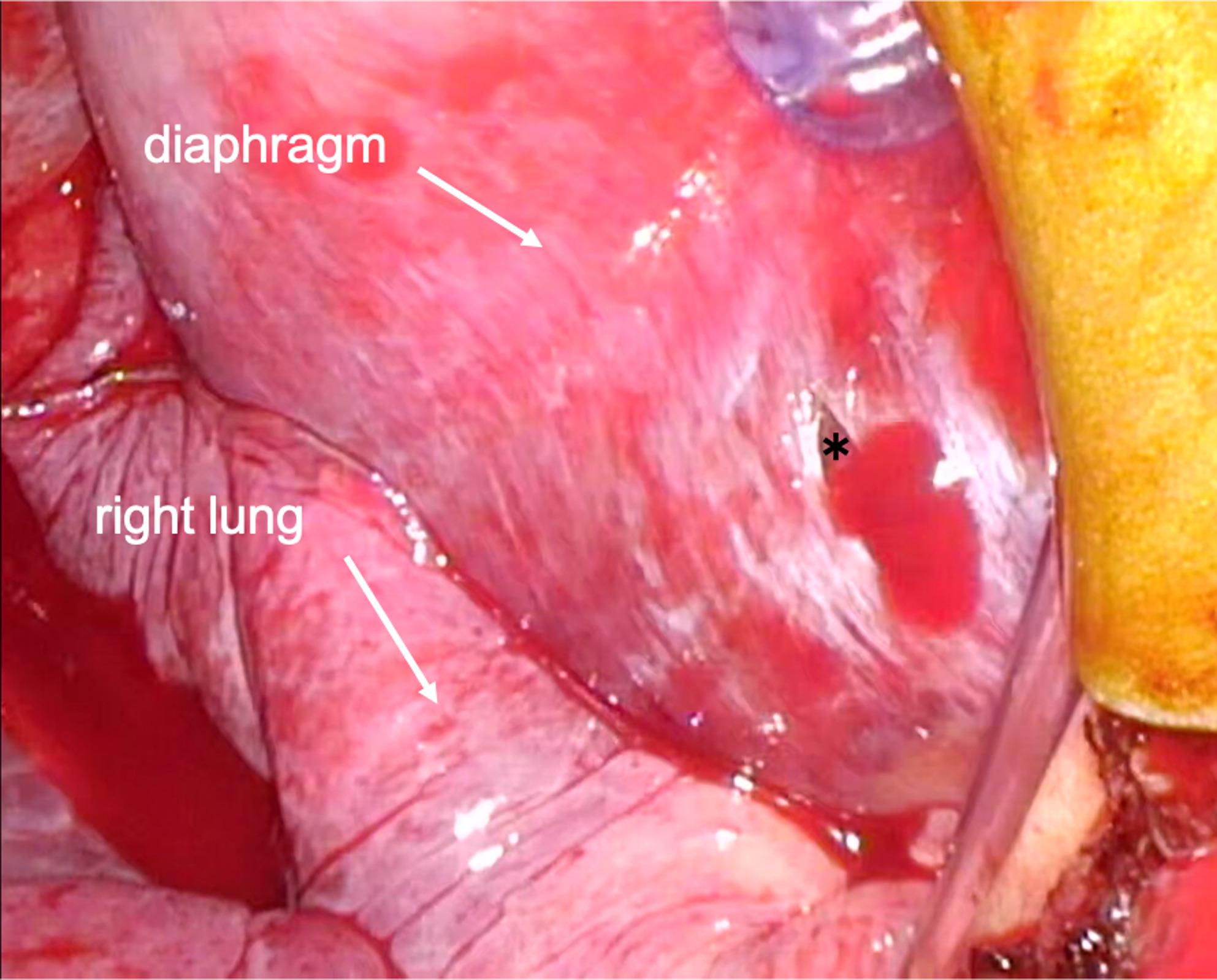
Laparoscopic view showing pinhole bleeding from the right diaphragm (*: pinhole)

**Fig. 3:**
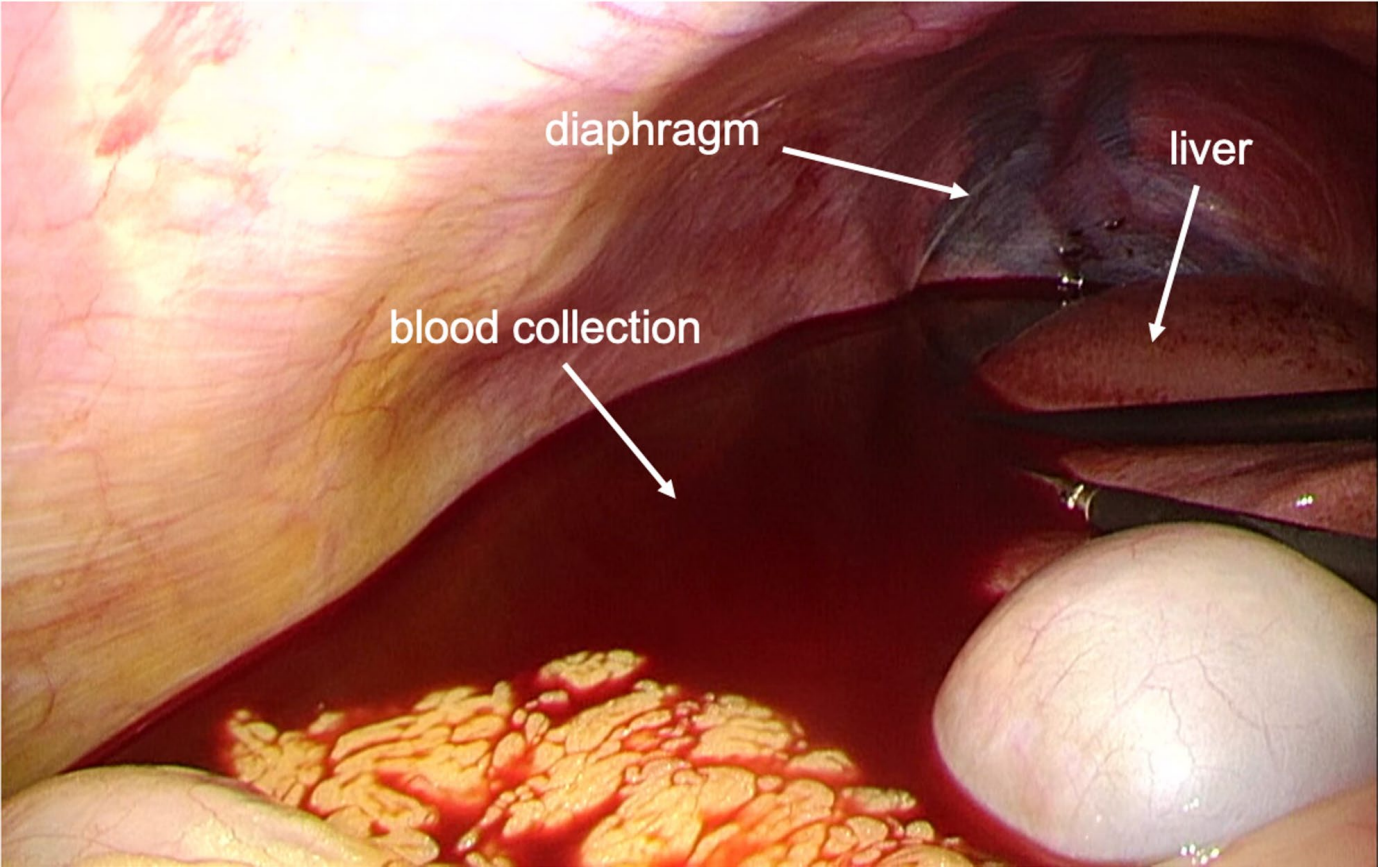
Laparoscopic view of blood collection around the liver

**Fig. 4:**
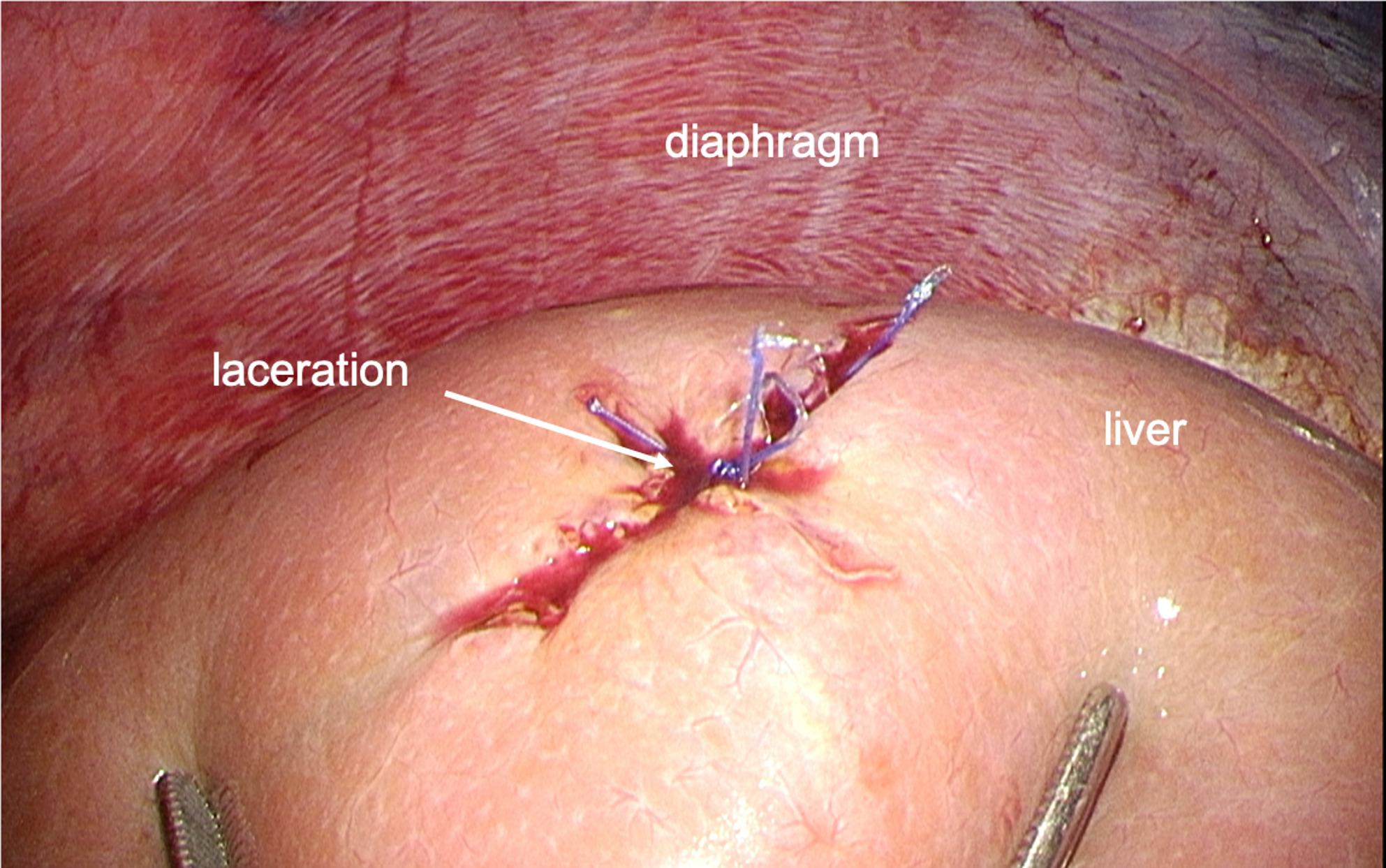
Laparoscopic view demonstrating an approsimatelry 1-cm laceration on the liver

The patient was scheduled to undergo mitral valvuloplasty (MVP), tricuspid annuloplasty (TAP), and the Maze procedure via a right mini-thoracotomy. After induction of general anesthesia and placement of invasive monitors, a bronchial blocker was inserted to facilitate one-lung ventilation and right lung collapse. The mini-thoracotomy was performed through a 7-cm right anterolateral incision. Cardiopulmonary bypass (CPB) was established via femoral arterial and venous cannulation through the right groin, which is the standard cannulation strategy for MICS mitral cases at our institution. The camera trocar (5-mm thoracoscopic port) was inserted without thoracoscopic visualization once the mini-thoracotomy incision and exposure were completed. No immediate bleeding was observed in the thoracic cavity. CPB was initiated uneventfully. During CPB, the perfusionist noted a progressive decline in reservoir volume of uncertain origin. No active bleeding was seen in the operative field, TEE confirmed correct positioning of the inferior and superior vena cava cannulas, and no fluid collection was detected in the chest cavity. Circuit volume and systemic perfusion were therefore maintained with ongoing replacement of packed red blood cells and crystalloids throughout CPB. Following separation from CPB, the patient required continuous volume repletion, although adequate surgical hemostasis had been achieved. While searching for other potential bleeding sources using TEE, deep transgastric TEE views subsequently revealed a hypoechoic space around the liver, suggestive of significant intra-abdominal fluid collection (Fig. 1). Simultaneously, pinhole bleeding from the right side of the diaphragm was noted (Fig. 2). A gastrointestinal surgical team was consulted, and an exploratory laparoscopy was performed. The procedure revealed blood collection surrounding the liver (Fig. 3), as well as an approximately 1-cm laceration on the liver surface (Fig. 4). Hemostasis was achieved by suturing the laceration, and no other source of bleeding was identified. The remainder of the surgery proceeded without complications, and the patient was transferred to the ICU without sequelae. He was extubated on postoperative day (POD) 1, and the subsequent postoperative course was uneventful.

## Discussion and Conclusions

This case is distinguished from prior reports of liver injury during MICS in two important ways. First, the mechanism of injury appears to be related to cranial displacement of the diaphragm following right-lung collapse and enlarged cirrhotic liver, which brought the liver into the trajectory of the camera trocar. This combination of diaphragmatic displacement and hepatomegaly underscores how patient-specific anatomy can transform an otherwise standard trocar insertion into a high-risk maneuver.

Retrospectively, the liver injury might have been recognized earlier had the gradual decline in CPB reservoir volume been attributed to intra-abdominal hemorrhage. However, reservoir volume can fall for various reasons during CPB, including intrathoracic bleeding, surgical manipulation, cannula malposition or kinking, or obstruction of blood flow due to airlock. In this case, the decline was gradual and was able to manage with volume supplementation. Thus, intra-abdominal hemorrhage was noticed only after separation from CPB.

Second, the timing of identifying bleeding source was different from the similar cases previously reported. In our case, liver laceration was detected intraoperatively by TEE, allowing timely laparoscopic intervention and avoidance of delayed postoperative diagnosis. Liver injury during MICS is rare; only a few cases have been reported, and all were diagnosed postoperatively [2, 3]. In these reports, liver laceration resulted either from a diaphragmatic suture during minithoracotomy or from contact between a robotic arm and the right hepatic lobe. The bleeding became apparent only hours after surgery when hemodynamic deterioration or abdominal signs developed, and patients required reoperation with worse outcomes [4]. By contrast, in our case the liver injury was successfully diagnosed intraoperatively using TEE.

MICS is performed through smaller incisions than sternotomy, limiting access to the surgical field. TEE is useful not only for confirming procedural success but also for identifying complications requiring immediate correction. TEE assists in the navigation of guidewires and placement of venous cannulas, both of which can contribute to liver or atrial injury. TEE is also useful for assessing valvular function, ventricular performance, intracardiac air, procedural adequacy, and unexpected events [5, 6]. Given the potential for unrecognized intra-abdominal bleeding, we believe that standard TEE protocols during MICS should consider including abdominal free fluid assessment.

From a preventive standpoint, careful trocar placement under direct thoracoscopic visualization, which was implemented in our institution after this event. Also, awareness of altered anatomy such as hepatomegaly or cranially displaced diaphragm, and close communication between the surgical and anesthesia teams are essential. Preoperative imaging to identify liver enlargement and intraoperative confirmation of trocar trajectory may help reduce the risk of unrecognized solid-organ injury.

Intra-abdominal bleeding during MICS can be difficult to detect early. In this case, TEE successfully identified subdiaphragmatic free fluid. Bleeding was successfully managed promptly with laparoscopic hemostasis. We recommend a routine deep transgastric sweep to assess intra-abdominal free fluid during intraoperative TEE in MICS.

## Data Availability

No datasets were generated or analysed during the current study.

## Abbreviations

CPB: Cardiopulmonary bypass
ICU: Intensive care unit
MICS: Minimally invasive cardiac surgery
MR: Mitral regurgitation
MVP: Mitral valvuloplasty
POD: Postoperative day
TAP: Tricuspid annuloplasty
TEE: Transesophageal echocardiography
TR: Tricuspid regurgitation

## References

[1] Dieberg G, Smart NA, King N. Minimally invasive cardiac surgery: a systematic review and meta-analysis. Int J Cardiol. 2016;223:554–60. 27557486 10.1016/j.ijcard.2016.08.227

[2] Hashim PW, Hashim SW. Liver laceration from a diaphragmatic suture in minithoracotomy mitral valve repair. J Card Surg. 2014;29(4):476–7. 24750236 10.1111/jocs.12346

[3] Duan J, Sun T, Ge S, Zhang C, Liu Z, Gong Q. A case of abdominal bleeding after mitral valvuloplasty assisted by da Vinci robotic surgery. J Card Surg. 2020;35(3):683–5. 31971268 10.1111/jocs.14413

[4] Kristensen KL, Rauer LJ, Mortensen PE, Kjeldsen BJ. Reoperation for bleeding in cardiac surgery. Interact Cardiovasc Thorac Surg. 2012;14(6):709–13. 22368106 10.1093/icvts/ivs050 PMC3352720

[5] Malik V, Hote M, Jha A. Minimally invasive cardiac surgery and transesophageal echocardiography. Ann Card Anaesth. 2014;17(2):125. 24732611 10.4103/0971-9784.129844

[6] Prempeh ABA, Scherman J, Swanevelder JL. Transesophageal echocardiography in minimally invasive cardiac surgery. Curr Opin Anaesthesiol. 2020;33(1):83–91. 31789893 10.1097/ACO.0000000000000807

